# Supplementary material for: STAT5A and STAT5B—Twins with Different Personalities in Hematopoiesis and Leukemia
Source: Cancers (Basel). 2019 Nov 4;11(11):1726. doi: 10.3390/cancers11111726 (PMC6895831; doi:10.3390/cancers11111726)
Supplement: Supplementary file 1 [file cancers-11-01726-s001.pdf]

Supplementary Materials

STAT5A and STAT5B—Twins with Different Personalities in Hematopoiesis and Leukemia

Barbara Maurer, Sebastian Kollmann, Judith Pickem, Andrea Hoelbl-Kovacic and Veronika Sexl

Figure S1

Human

```
Length: 804
Identity:     732/804 (91.0%)
Similarity:   748/804 (93.0%)
Gaps:          27/804 ( 3.4%)
Score: 3787.0

':' for a similarity which scores more than 1.0
'.' for any small positive score
'|' for an identity where both sequences have the same residue regardless
of its score
space for a mismatch or a gap
```

Oligomerisation domain
Coiled-coil domain
DNA binding domain
Linker domain
SH2 domain
Transactivation domain
Phosphorlyation
Acetylation
Methylation
O-GlcNAc

```
STA5A_HUMAN         1 MAGWIQAQQLQGDALRQMQVLYGQHFPIEVRHYLAQWIESQPWDAIDLDN
50
                      ||.|||||||||:||.|||.||||||||||||||:||||||.||::||||
STA5B_HUMAN         1 MAVWIQAQQLQGEALHQMQALYGQHFPIEVRHYLSQWIESQAWDSVDLDN
50

STA5A_HUMAN        51 PQDRAQATQLLEGLVQELQKKAEHQVGEDGFLLKIKLGHYATQLQKTYDR
100
                      ||:..:|||||||||||||||||||||||||||||||||||||||.||||
STA5B_HUMAN        51 PQENIKATQLLEGLVQELQKKAEHQVGEDGFLLKIKLGHYATQLQNTYDR
100

STA5A_HUMAN       101 CPLELVRCIRHILYNEQRLVREANNCSSPAGILVDAMSQKHLQINQTFEE
150
                      ||:|||||||||||||||||||||||.||||.|.||||||||||||||||
STA5B_HUMAN       101 CPMELVRCIRHILYNEQRLVREANNGSSPAGSLADAMSQKHLQINQTFEE
150

STA5A_HUMAN       151 LRLVTQDTENELKKLQQTQEYFIIQYQESLRIQAQFAQLAQLSPQERLSR
200
                      ||||||||||||||||||||||||||||||||||||..||||||||||||
STA5B_HUMAN       151 LRLVTQDTENELKKLQQTQEYFIIQYQESLRIQAQFGPLAQLSPQERLSR
200
```

```
STA5A_HUMAN        201 ETALQQKQVSLEAWLQREAQTLQQYRVELAEKHQKTLQLLRKQQTIILDD    250
                       ||||||||||||||||||||||||||||||||||||||||||||||||||
STA5B_HUMAN        201 ETALQQKQVSLEAWLQREAQTLQQYRVELAEKHQKTLQLLRKQQTIILDD    250

STA5A_HUMAN        251 ELIQWKRRQQLAGNGGPPEGSLDVLQSWCEKLAEIIWQNRQQIRRAEHLC    300
                       ||||||||||||||||||||||||||||||||||||||||||||||||||
STA5B_HUMAN        251 ELIQWKRRQQLAGNGGPPEGSLDVLQSWCEKLAEIIWQNRQQIRRAEHLC    300

STA5A_HUMAN        301 QQLPIPGPVEEMLAEVNATITDIISALVTSTFIIEKQPPQVLKTQTKFAA    350
                       ||||||||||||||||||||||||||||||||||||||||||||||||||
STA5B_HUMAN        301 QQLPIPGPVEEMLAEVNATITDIISALVTSTFIIEKQPPQVLKTQTKFAA    350

STA5A_HUMAN        351 TVRLLVGGKLNVHMNPPQVKATIISEQQAKSLLKNENTRNECSGEILNNC    400
                       ||||||||||||||||||||||||||||||||||||||||:.||||||||
STA5B_HUMAN        351 TVRLLVGGKLNVHMNPPQVKATIISEQQAKSLLKNENTRNDYSGEILNNC    400

STA5A_HUMAN        401 CVMEYHQATGTLSAHFRNMSLKRIKRADRRGAESVTEEKFTVLFESQFSV    450
                       ||||||||||||||||||||||||||:||||||||||||||:||||||||
STA5B_HUMAN        401 CVMEYHQATGTLSAHFRNMSLKRIKRSDRRGAESVTEEKFTILFESQFSV    450

STA5A_HUMAN        451 GSNELVFQVKTLSLPVVVIVHGSQDHNATATVLWDNAFAEPGRVPFAVPD    500
                       |.|||||||||||||||||||||||:||||||||||||||||||||||||
STA5B_HUMAN        451 GGNELVFQVKTLSLPVVVIVHGSQDNNATATVLWDNAFAEPGRVPFAVPD    500

STA5A_HUMAN        501 KVLWPQLCEALNMKFKAEVQSNRGLTKENLVFLAQKLFNNSSSHLEDYSG    550
                       ||||||||||||||||||||||||||||||||||||||||||||||||||
STA5B_HUMAN        501 KVLWPQLCEALNMKFKAEVQSNRGLTKENLVFLAQKLFNNSSSHLEDYSG    550

STA5A_HUMAN        551 LSVSWSQFNRENLPGWNYTFWQWFDGVMEVLKKHHKPHWNDGAILGFVNK    600
                       |||||||||||||||.||||||||||||||||||.|||||||||||||||
STA5B_HUMAN        551 LSVSWSQFNRENLPGRNYTFWQWFDGVMEVLKKHLKPHWNDGAILGFVNK    600

STA5A_HUMAN        601 QQAHDLLINKPDGTFLLRFSDSEIGGITIAWKFDSPERNLWNLKPFTTRD    650
                       |||||||||||||||||||||||||||||||||||.||..|||.||||||
STA5B_HUMAN        601 QQAHDLLINKPDGTFLLRFSDSEIGGITIAWKFDSQERMFWNLMPFTTRD    650

STA5A_HUMAN        651 FSIRSLADRLGDLSYLIYVFPDRPKDEVFSKYYTPV-----LAKAVDGYV    695
                       |||||||||||||:||||||||||||||:|||||||     .||||||||
STA5B_HUMAN        651 FSIRSLADRLGDLNYLIYVFPDRPKDEVYSKYYTPVPCESATAKAVDGYV    700

STA5A_HUMAN        696 KPQIKQVVPEFVNASADAGGSSATYMDQAPSPAVCPQAPYNMYPQNPDHV    745
```

```
                    ||||||||||||||||||||.||||||||||||||||.||||||||||.|
STA5B_HUMAN       701
KPQIKQVVPEFVNASADAGGGSATYMDQAPSPAVCPQAHYNMYPQNPDSV750

STA5A_HUMAN       746 LDQDGEFDLDETMDVARHVEELLRRPMDS-----LDSRLSPPAGLFTSAR
790
                      ||.||:|||::||||||.|||||.|||||     ..|
STA5B_HUMAN       751 LDTDGDFDLEDTMDVARRVEELLGRPMDSQWIPHAQS-------------
787

STA5A_HUMAN       791 GSLS     794

STA5B_HUMAN       788 ----     787
```

Murine

```
# Length: 798
# Identity:     730/798 (91.5%)
# Similarity:   749/798 (93.9%)
# Gaps:          17/798 ( 2.1%)
# Score: 3787.0

':' for a similarity which scores more than 1.0
'.' for any small positive score
'|' for an identity where both sequences have the same residue regardless
of its score
space for a mismatch or a gap
```

Oligomerisation domain
Coiled-coil domain
DNA binding domain
Linker domain
SH2 domain
Transactivation domain
Phosphorlyation
Acetylation
Methylation
O-GlcNAc

```
STA5A_MOUSE         1 MAGWIQAQQLQGDALRQMQVLYGQHFPIEVRHYLAQWIESQPWDAIDLDN
50
                      ||.||||||||||||.|||.||||||||||||||:||||||.||:|||||
STA5B_MOUSE         1 MAMWIQAQQLQGDALHQMQALYGQHFPIEVRHYLSQWIESQAWDSIDLDN
50

STA5A_MOUSE        51 PQDRGQATQLLEGLVQELQKKAEHQVGEDGFLLKIKLGHYATQLQNTYDR
100
                      ||:..:|||||||||||||||||||||||||||||||||||||||:||||
STA5B_MOUSE        51 PQENIKATQLLEGLVQELQKKAEHQVGEDGFLLKIKLGHYATQLQSTYDR
100

STA5A_MOUSE       101 CPMELVRCIRHILYNEQRLVREANNCSSPAGVLVDAMSQKHLQINQRFEE
150
                      |||||||||||||||||||||||||.|||||.|.||||||||||||.|||
STA5B_MOUSE       101 CPMELVRCIRHILYNEQRLVREANNGSSPAGSLADAMSQKHLQINQTFEE
150
```

```
STA5A_MOUSE      151 LRLITQDTENELKKLQQTQEYFIIQYQESLRIQAQFAQLGQLNPQERMSR
200
                     ||||||||||||||||||||||||||||||||||||||||||||||||||
STA5B_MOUSE      151 LRLITQDTENELKKLQQTQEYFIIQYQESLRIQAQFAQLGQLNPQERMSR
200

STA5A_MOUSE      201 ETALQQKQVSLETWLQREAQTLQQYRVELAEKHQKTLQLLRKQQTIILDD
250
                     ||||||||||||||||||||||||||||||||||||||||||||||||||
STA5B_MOUSE      201 ETALQQKQVSLETWLQREAQTLQQYRVELAEKHQKTLQLLRKQQTIILDD
250

STA5A_MOUSE      251 ELIQWKRRQQLAGNGGPPEGSLDVLQSWCEKLAEIIWQNRQQIRRAEHLC
300
                     ||||||||||||||||||||||||||||||||||||||||||||||||||
STA5B_MOUSE      251 ELIQWKRRQQLAGNGGPPEGSLDVLQSWCEKLAEIIWQNRQQIRRAEHLC
300

STA5A_MOUSE      301 QQLPIPGPVEEMLAEVNATITDIISALVTSTFIIEKQPPQVLKTQTKFAA
350
                     ||||||||||||||||||||||||||||||||||||||||||||||||||
STA5B_MOUSE      301 QQLPIPGPVEEMLAEVNATITDIISALVTSTFIIEKQPPQVLKTQTKFAA
350

STA5A_MOUSE      351 TVRLLVGGKLNVHMNPPQVKATIISEQQAKSLLKNENTRNECSGEILNNC
400
                     ||||||||||||||||||||||||||||||||||||||||:.||||||||
STA5B_MOUSE      351 TVRLLVGGKLNVHMNPPQVKATIISEQQAKSLLKNENTRNDYSGEILNNC
400

STA5A_MOUSE      401 CVMEYHQATGTLSAHFRNMSLKRIKRADRRGAESVTEEKFTVLFESQFSV
450
                     ||||||||||||||||||||||||||:||||||||||||||:||:|||||
STA5B_MOUSE      401 CVMEYHQATGTLSAHFRNMSLKRIKRSDRRGAESVTEEKFTILFDSQFSV
450

STA5A_MOUSE      451 GSNELVFQVKTLSLPVVVIVHGSQDHNATATVLWDNAFAEPGRVPFAVPD
500
                     |.|||||||||||||||||||||||:||||||||||||||||||||||||
STA5B_MOUSE      451 GGNELVFQVKTLSLPVVVIVHGSQDNNATATVLWDNAFAEPGRVPFAVPD
500

STA5A_MOUSE      501 KVLWPQLCEALNMKFKAEVQSNRGLTKENLVFLAQKLFNISSNHLEDYNS
550
                     ||||||||||||||||||||||||||||||||||||||||||||||||||
STA5B_MOUSE      501 KVLWPQLCEALNMKFKAEVQSNRGLTKENLVFLAQKLFNISSNHLEDYNS
550

STA5A_MOUSE      551 MSVSWSQFNRENLPGWNYTFWQWFDGVMEVLKKHHKPHWNDGAILGFVNK
600
                     |||||||||||||||.||||||||||||||||||.|||||||||||||||
STA5B_MOUSE      551 MSVSWSQFNRENLPGRNYTFWQWFDGVMEVLKKHLKPHWNDGAILGFVNK
600

STA5A_MOUSE      601 QQAHDLLINKPDGTFLLRFSDSEIGGITIAWKFDSPDRNLWNLKPFTTRD
650
                     |||||||||||||||||||||||||||||||||||.:|..|||.||||||
STA5B_MOUSE      601 QQAHDLLINKPDGTFLLRFSDSEIGGITIAWKFDSQERMFWNLMPFTTRD
650

STA5A_MOUSE      651 FSIRSLADRLGDLNYLIYVFPDRPKDEVFAKYYTPV-----LAKAVDGYV
695
                     ||||||||||||||||||||||||||||::||||||     .|||.||||
```

```
STA5B_MOUSE       651 FSIRSLADRLGDLNYLIYVFPDRPKDEVYSKYYTPVPCEPATAKAADGYV
700

STA5A_MOUSE       696 KPQIKQVVPEFVNASTDAGASATYMDQAPSPVVCPQPHYNMYPPNPDPVL
745
                      |||||||||||.|||||||:.|||||||||||||||.||||||||||.||
STA5B_MOUSE       701 KPQIKQVVPEFANASTDAGSGATYMDQAPSPVVCPQAHYNMYPPNPDSVL
750

STA5A_MOUSE       746 DQDGEFDLDESMDVARHVEELLRRPMDSLDARLSPPAGLFTSARSSLS
793
                      |.||:|||:::|||||.|||||.|||||   :..|.|..
STA5B_MOUSE       751 DTDGDFDLEDTMDVARRVEELLGRPMDS---QWIPHAQS---------
786
```

Figure S1. Protein alignment of human or murine STAT5A and STAT5B. Alignment and visualization were done using EMBL-EBI search and sequence analysis tools [1].

References

1. Madeira, F.; Park, Y.m.; Lee, J.; Buso, N.; Gur, T.; Madhusoodanan, N.; Basutkar, P.; Tivey, A.R.N.; Potter, S.C.; Finn, R.D., et al. The EMBL-EBI search and sequence analysis tools APIs in 2019. *Nucleic Acids Res.* **2019**, *47*, W636–W641, doi:10.1093/nar/gkz268.
